# Lunar and mars gravity induce similar changes in spinal motor control as microgravity

**DOI:** 10.3389/fphys.2023.1196929

**Published:** 2023-07-26

**Authors:** Jaap Swanenburg, Christopher A. Easthope, Anita Meinke, Anke Langenfeld, David A. Green, Petra Schweinhardt

**Affiliations:** ^1^ Department of Chiropractic Medicine, Integrative Spinal Research ISR, Balgrist University Hospital, Zürich, Switzerland; ^2^ Faculty of Medicine, Institute of Anatomy, University of Zurich, Zurich, Switzerland; ^3^ Innovation Cluster Space and Aviation (UZH Space Hub), Air Force Center, University of Zurich, Dübendorf, Switzerland; ^4^ Cereneo—Center for Interdisciplinary Research, Vitznau, Switzerland; ^5^ Lake Lucerne Institute, Vitznau, Switzerland; ^6^ Centre of Human and Applied Physiological Sciences, King’s College London, London, United Kingdom; ^7^ Space Medicine Team, European Astronaut Centre, European Space Agency, Cologne, Germany; ^8^ KBRwyle GmbH, Cologne, Germany

**Keywords:** stiffness, spine, microgravity, hypergravity, lunar, lumbar, parabolic flight

## Abstract

**Introduction:** Once more, plans are underway to send humans to the Moon or possibly even to Mars. It is therefore, important to know potential physiological effects of a prolonged stay in space and to minimize possible health risks to astronauts. It has been shown that spinal motor control strategies change during microgravity induced by parabolic flight. The way in which spinal motor control strategies change during partial microgravity, such as that encountered on the Moon and on Mars, is not known.

**Methods:** Spinal motor control measurements were performed during Earth, lunar, Mars, and micro-gravity conditions and two hypergravity conditions of a parabola. Three proxy measures of spinal motor control were recorded: spinal stiffness of lumbar L3 vertebra using the impulse response, muscle activity of lumbar flexors and extensors using surface electromyography, and lumbar curvature using two curvature distance sensors placed at the upper and lower lumbar spine. The participants were six females and six males, with a mean age of 33 years (standard deviation: 7 years).

**Results:** Gravity condition had a statistically significant (Friedmann tests) effect spinal stiffness (*p* < 0.001); on EMG measures (multifidus (*p* = 0.047), transversus abdominis (*p* < 0.001), and psoas (*p* < 0.001) muscles) and on upper lumbar curvature sensor (*p* < 0.001). No effect was found on the erector spinae muscle (*p* = 0.063) or lower curvature sensor (*p* = 0.170). Post hoc tests revealed a significant increase in stiffness under micro-, lunar-, and Martian gravity conditions (all *p’s* < 0.034). Spinal stiffness decreased under both hypergravity conditions (all *p’s* ≤ 0.012) and decreased during the second hypergravity compared to the first hypergravity condition (*p* = 0.012).

**Discussion:** Micro-, lunar-, and Martian gravity conditions resulted in similar increases in spinal stiffness, a decrease in transversus abdominis muscle activity, with no change in psoas muscle activity and thus modulation of spinal motor stabilization strategy compared to those observed under Earth’s gravity. These findings suggest that the spine is highly sensitive to gravity transitions but that Lunar and Martian gravity are below that required for normal modulation of spinal motor stabilization strategy and thus may be associated with LBP and/or IVD risk without the definition of countermeasures.

## 1 Introduction

Back pain and herniated intervertebral discs (IVDs) are not only a global burden but also a problem for astronauts after long duration missions in microgravity (µg) (Nieminen et al., 2021; Bailey et al., 2022). Astronauts have an increased risk of back pain and IVD herniation within 1 year following long space missions (Johnston et al., 2010; Young et al., 2011; Pool-Goudzwaard et al., 2015). Various causes of low back pain (LBP) and/or increased risk of IVD herniation during and after a space mission have been hypothesized in recent years (Wing et al., 1991; Pool-Goudzwaard et al., 2015). In particular, swelling of the IVDs (Sayson and Hargens, 2008), reduced para-spinal muscle tone (Chang et al., 2016; McNamara et al., 2019), spinal curvature flattening, and altered spinal motor control during and after a space mission have been observed (Andreoni et al., 2000).

Recent studies have shown that changes in IVD height and hydration under µg conditions are negligible (Bailey et al., 2018; Bailey et al., 2022). Altered spinal motor control can be a serious problem for astronauts returning from space missions or to successfully complete any future missions to the Moon or Mars (Green and Scott, 2018). Spinal motor control is an essential stabilization mechanism for human spinal function in daily life (Mergner and Rosemeier, 1998; Roijezon et al., 2015; Treleaven, 2017). Motor control consists of active (muscles), passive (bones, discs, joints, and ligaments), and the neural motor control subsystems (Panjabi, 1992b). The neural motor control subsystem obtains information from the active and passive subsystems, which are used by the neural subsystem to stabilize the spine (Panjabi, 1992a; Panjabi, 1992b).

Muscle viscoelastic properties such as tone, including of the erector spinae, have been identified to be modulated during parabolic flight (Schneider et al., 2015) and 3-day Dry Immersion—a ground-based model of µg that is associated with the induction of back pain (Treffel et al., 2017). However, the functional value of such changes remains to be determined (Plehuna et al., 2022). Yet in another ground-based model of µg termed hyper-buoyancy flotation (HBF) developed to model stature elongation and back pain (Green et al., 2023) is also associated with modulation in lumbar kinematics (Breen et al., 2023). Recent parabolic flight studies have also showed a rapid increase in lumbar (L3) spine stiffness, defined as resistance to deformation of the spinal system (Panjabi, 1992b; Panjabi, 1992a; Hodges et al., 2013; Needle et al., 2014). Spinal stiffness is a proxy measure of spinal motor control at transient μg (approximately 20 s) (Swanenburg et al., 2018; Swanenburg et al., 2020; Glaus et al., 2021). However, equivalent data in hypo and hypergravity have yet to be evaluation.

A similar decrease in lumbar and thoracic spinal stiffness was shown in a study on 100 healthy young adults while carrying a load equal to 50% of the subject’s body weight on their shoulders (Hausler et al., 2020). In a later study, the authors examined the change in spinal stiffness with a gradual increase in axial load reporting that at an axial load of ≥45% of the participant’s body weight, spinal stiffness decreased (Glaus et al., 2021). Also, active and passive thoracic spinal stiffness was found to be decreased during trunk exercises under “artificial gravity”, without a change in cervical or lumbar spinal stiffness (De Martino et al., 2020). Together, the results of these studies demonstrate the adaptability and complexity of spinal motor control strategies and the influence of differences in gravity and axial loading conditions.

Humans will in the near future once again encounter the challenges of operating in hypogravity when returning to the Lunar surface and subsequently Mars. However, it is unknown if, or how, spinal motor control strategies are modulated under Lunar or Martian gravity conditions. Given the potential mission critical implications of LBP and/or IVD herniation on the Lunar or Martian surface it is vital to understand and potentially define countermeasures to mitigate the effects of hypogravity with larger operational constraints compared to the International Space Station (Scott et al., 2019).

Therefore, the objective of the current study was to determine the response of spinal motor control (via changes in lumbar spinal stiffness, muscle activity, and lumbar curvature) to µg, lunar and Mars hypogravity, and hypergravity.

## 2 Methods

### 2.1 Participants and parabolic flight

Twelve healthy individuals (mean age: 33 years ±7 years; six females) with no acute LBP participated in this study. The participants passed required aviation medical screening during which neural or musculoskeletal disorders were excluded (Ullrich and Buhler, 2019). The spinal motor control measurements were conducted during the European Space Agency’s (ESA) 74th and 76th partial gravity parabolic flight campaigns (PFCs) in Paderborn, Germany operated by Novespace (Bordeaux, France) on an Airbus A310 ZERO-G. All participants provided written, informed consent prior to inclusion in the study. The French “Comite de protection des personnes EST-III” approved the study (Nr-ID-RCB: 2018-A011294-51/Nr-CPP: 18.06.09). To prevent motion sickness, all participants were given scopolamine (0.25 mg/1 mL; 0.7 mL for males and 0.5 mL for females) 30 min before the flight (Spinks and Wasiak, 2011; Ritzmann et al., 2016).

### 2.2 Experimental design

**FIGURE 1:**
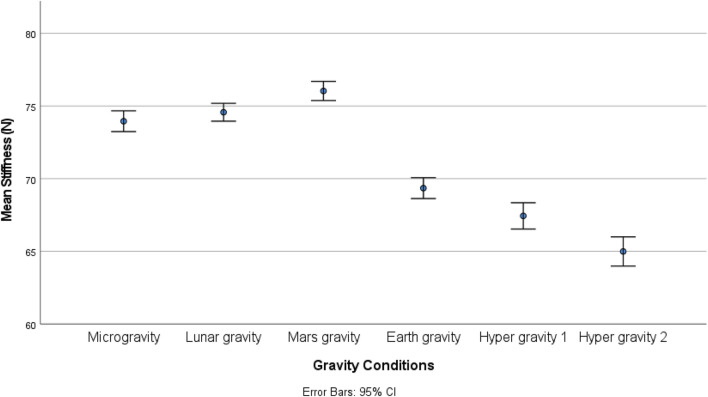
Trajectories flown during parabolic flight; microgravity, lunar gravity, and Mars gravity.

Spinal motor control parameters were assessed during µg, lunar gravity (0.16 g; lunar-g), Martian gravity (0.36 g; Mars-g), Earth’s gravity (1 g), and hypergravity (1.8 g) during the parabolic flights. Trajectories flown during parabolic flight; microgravity, lunar gravity, and Mars gravity are shown in Figure 1. During each of the two PFCs, three flights were performed. Each flight included two sequences of 15 parabolas, consisting of 5 µg, five lunar-g, and five Mars-g gravity conditions following a single µg familiarization parabola. The order of lunar, Mars and Micro gravity was changed for each flight. Each parabola started with a horizontal flight with Earth’s gravity, followed by a steep climb flight that induced hypergravity (hyper-g-1). When a sufficient upward velocity is reached, the pilots “push-over” and reduce thrust so µg is achieved; aircraft and occupants together fall at 9.81 m/s^2^ (Karmali and Shelhamer, 2008). Subsequently, there was a second hypergravity (hyper-g-2) phase, followed by a return to normal level flight. In addition, during the flight the order of gravity levels (each 5 parabolas) was reversed after 15 parabolas. The hyper-g-2 phase was used to examine possible G-transition effects caused by the rapid changes in gravitoinertial forces (Cheung and Bateman, 2001). Measurements under different gravity conditions.

**FIGURE 2:**
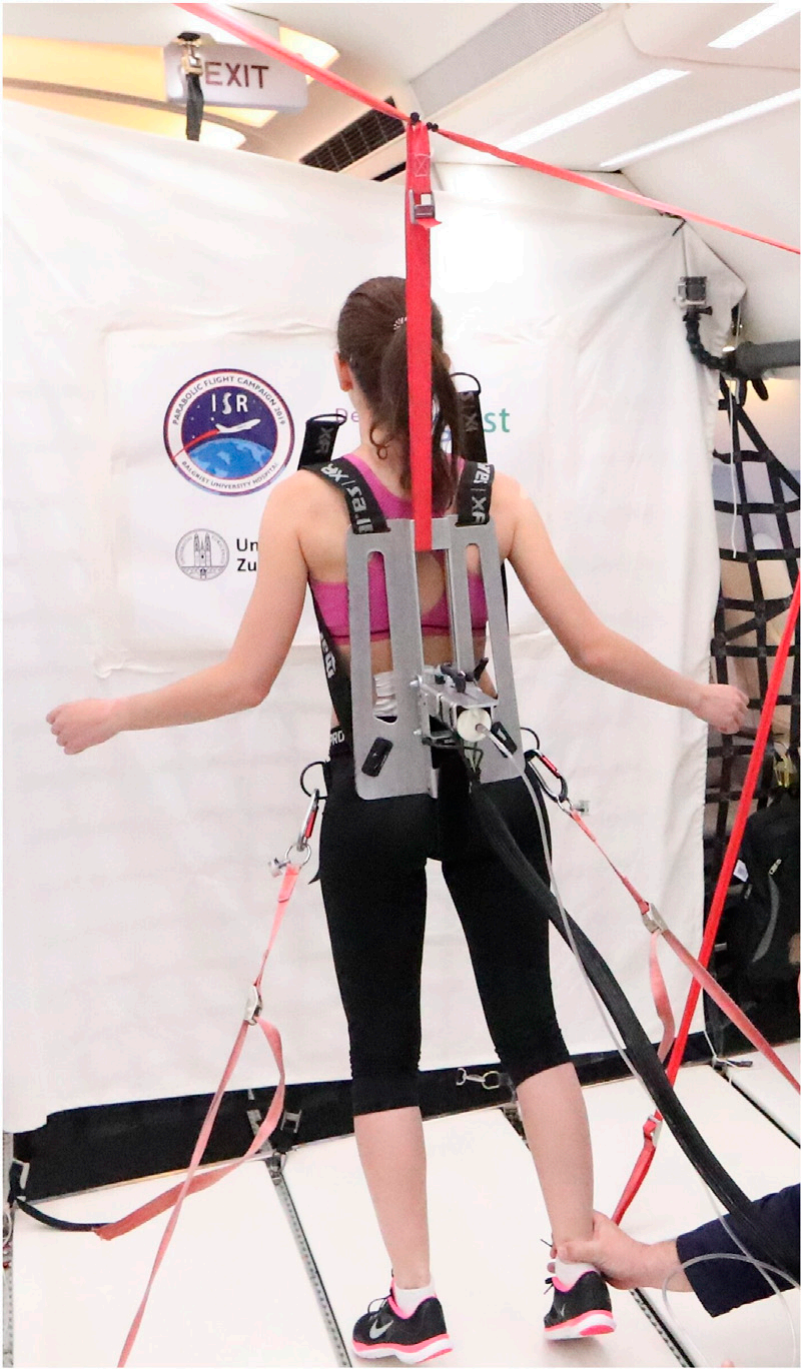
Measurements in microgravity during the 76st ESA Parabolic Flight Campaign 2021. Pic by Novespace.

The stiffness measuring device was mounted on a backplate that was strapped to the participants with a full-body harness to prevent loss of contact between the device and the participant during the different gravity conditions. In addition, each participant was tethered to the inside of the aircraft with ropes attached to the full-body harness to prevent them from falling in hypergravity (Swanenburg et al., 2018; Swanenburg et al., 2020). No data were collected during the first parabola, which was used to allow participants to familiarize themselves with the different gravity conditions. Also, no data were collected during the last parabola due to possible participant fatigue (Swanenburg et al., 2020). As breathing can affect the measurement of spinal stiffness, participants were instructed to hold their breath at the end of a normal exhalation before the measurement was commenced (Shirley et al., 2003). Figure 2 shows test subject wearing the full-body harness during parabolic flight.

### 2.3 Measurement setup

#### 2.3.1 Spinal stiffness

**FIGURE 3:**
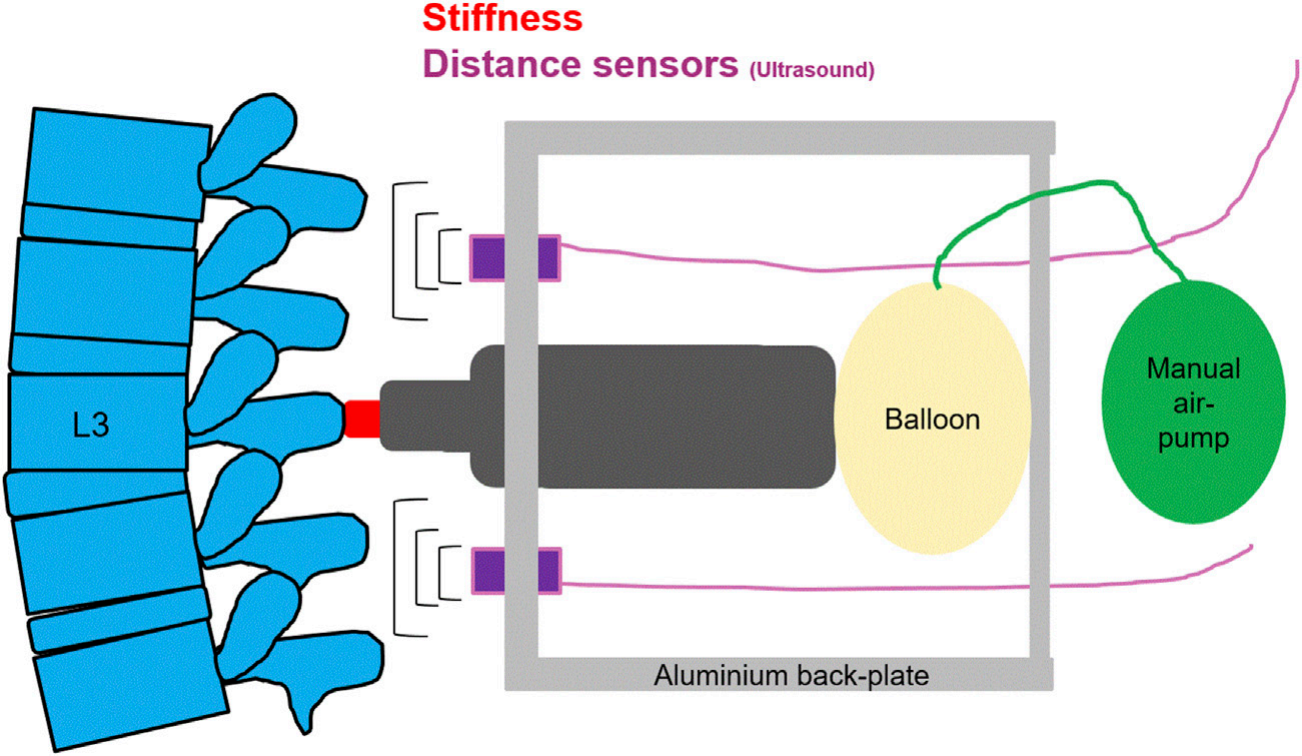
A schematic of the measurement set-up, for spinal stiffness, and lumbar curvature with two distance sensors.

A computerized analysis device, PulStar (Function Recording and Analysis System device PulStarFRAS; Sense Technology Inc., Halifax, PA, United States), was used to measure posterior-to-anterior spinal stiffness (Leach et al., 2003; Hofstetter et al., 2018; Hausler et al., 2020). An impulse head impactor was mounted on an aluminum plate, and an 80 N pulse was applied to the spinous process of L3 (Swanenburg et al., 2018). A preload of 18 N was required to trigger the impulse and to minimize the influence of soft tissue components like skin and subcutaneous layer between the impulse head and the spinous process (Leach et al., 2003; Swanenburg et al., 2020). Using a manual air pump and a balloon behind the pulse head, the required preload was created without touching the participant. The impulse response (spinal stiffness) quantifies the reaction of the muscles, joints, and ligaments to the energy applied by the impulse (Leach et al., 2003; Swanenburg et al., 2020). This response can be approximated using a linear, time-invariant system that is disturbed by a very short (< 1 m) input signal (impulse). Therefore, the impulse response can be expressed as a force (Newton) without time change (Girod et al., 2003; Swanenburg et al., 2020). Figure 3 shows schematic of the measurement set-up.

#### 2.3.2 Muscle activity

The human spine contains global and local muscle systems. The global system comprises muscles that connect the pelvis and thorax. The local system consists of muscles that attach or originate at the lumbar vertebrae (Bergmark, 1989). In this study, in the global muscle system, the *transversus abdominis muscle* was assessed. In the local muscle system, the *erector spinae* and *multifidi muscles* were assessed. *Psoas muscle* activity was also measured, because this is thought to increase lower lumbar spinal stiffness (Cholewicki and McGill, 1996; Juker et al., 1998). Wireless surface electromyography (EMG) transmitters (pico/aktos; Myon AG, Schwarzenberg, Switzerland) with integrated accelerometers were used to record muscle activity (McGill et al., 1996; Jiroumaru et al., 2014). A laptop recorded prefiltered EMG (2,000 Hz, bandpass: 10–500 Hz) and accelerometer (148 Hz, bandpass: 1–70 Hz) signals. Subject preparation and electrode placement were in accordance with to the Surface ElectroMyoGraphy for the Non-Invasive Assessment of Muscles (SENIAM) guidelines. The electrodes for the psoas muscle were located between the inguinal region, the sartorius muscle, and the femoral neurovascular bundle (Katsavrias et al., 2005). The readings of the accelerometers within the transmitters were used to confirm simulated gravitational forces during the PFC experiment.

#### 2.3.3 Lumbar curvature

Lumbar curvature was assessed using two ultrasonic distance sensors mounted on the aluminum Backplate of the full-body harness measuring the distance between the Backplate and the skin of the participant (Swanenburg et al., 2020). These ultrasonic distance sensors (UC250-F77-IU-IO-V31; Pepperl + Fuchs, Mannheim, Germany) were mounted on the aluminum Backplate at + 4 cm rostrally (upper sensor) and −4 cm caudally (lower sensor) to the stiffness measurement device (Swanenburg et al., 2020). The distance data were recorded continuously throughout the flight with 140 Hz and stored on a laptop**.**

### 2.4 Data processing

Muscle activity data was processed as described in our previous manuscript on this topic (Swanenburg et al., 2020). Briefly: Gravitational steady states were segmented using acceleration traces logged in the erector spinae EMG sensors ( µg , −0.1–0.1 × *g*; lunar-g, 0.08–0.28; Mars-g, 0.26–0.46; Earth-g, 0.9–1.1 × *g*; and hyper-g, 1.7–1.9 × *g*). An EMG extraction window of 12 s was used to mitigate gravitational transition effects at the boundaries of the steady state. Within the steady state, windows were shifted algorithmically to maximize signal-to-noise ratio and subsequently visually verified by an experienced operator to ensure that there was no artifact contamination of the analyzed EMG segment. Root Mean Square (RMS) values were calculated for each EMG segment and muscle and were normalized to baseline (Earth-g) for each parabola. The normalized RMS values were subsequently averaged for the left and right side and across parabolas.

Distance data describing lumbar curvature was cleaned and aggregated in R v 4.1.3 and MATLAB (2020b, Mathworks, Natick, MA, United States). Thresholds were used to remove outliers from the records. These thresholds were defined individually for each participant and ranged between 1.5 and 6 times the standard deviation above the mean of the record. In addition, aberrant values were removed from the records of each 4 participants for the upper and the lower sensor, where this seemed to improve the data quality. In some cases, the sensors had returned constant values- Therefore the complete distance data of the upper sensor were excluded from analysis for 3 participants and of the lower sensor for 1 participant. Distance data of only the lunar gravity parabolas were excluded for two participants. Means were calculated within the gravitational states described above and aggregated across parabolas for each participant.

### 2.5 Data analysis

Because of the small sample size, a non-parametric approach was used. Friedman’s two-way analysis of variance by rank was used to evaluate the effect of different gravity conditions on spinal stiffness, EMG, and distance sensor measurements distributions with Wilcoxon’s rank testing used for post-hoc analysis between each gravity condition significance assumed at *p* < 0.05. Bonferroni correction for multiple comparisons was used for EMG measurements of the four muscles (*p* < 0.0125). For the distance sensor measurements, *p* < 0.025 was considered significant. Statistical analysis was performed using the IBM SPSS version 25 (IBM SPSS Statistics for Windows, IBM Corp., Armonk, NY).

## 3 Results

### 3.1 Spinal stiffness

**FIGURE 4:**
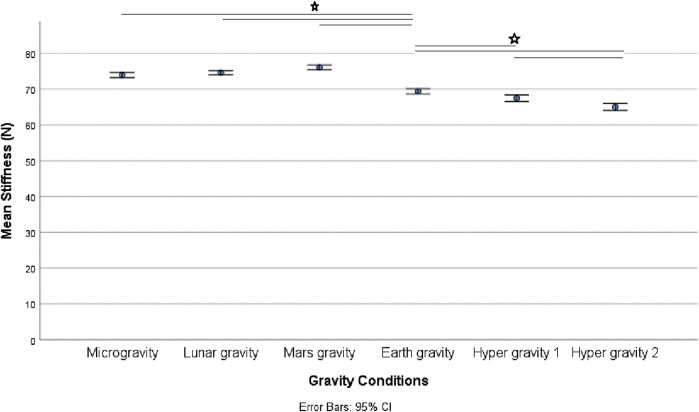
Mean spinal stiffness among the different gravity conditions.

There was a statistically significant (χ25) = 34.190; *p* < 0.001) different distribution of spinal stiffness among the different gravity conditions. The *post hoc* analysis revealed a significant increase in spinal stiffness during µg, lunar-g, and Mars-g compared to Earth-g, and a significant decrease in spinal stiffness during both hyper-g conditions vs Earth-g. No difference in spinal stiffness between µg, lunar-g, and Mars-g was observed. Hyper-g-2 resulted in significantly lower spinal stiffness than Hyper-g-1. Results are shown in Table 2 and Figure 4.

### 3.2 Muscle activity

The EMG data of 11 subjects were analyzed as the data from one subject were incomplete. A significant effect of gravity was observed in two of the four recorded muscles: the transversus abdominis, (χ25) = 42.740; *p* < 0.001), and psoas (χ25) = 29.026; *p* < 0.001). But not in the erector spinae (χ25) = 10.457, *p* = 0.063), multifidi (χ25) = 11.208, *p* = 0.047). Post hoc analysis showed a significant decrease in transversus abdominis muscle activity during µg (*p* = 0.006) and Mars-g (0.008) conditions and a significant increase during both hyper-g phases (*p* = 0.003). Psoas muscle activity increased significantly during both hyper-g phases (*p* = 0.003 and *p* = 0.004, respectively) and during the second hyper-g-2 phase (*p* = 0.003) compared to that in the first hyper-g-2 phase.

### 3.3 Lumbar curvature

Friedman’s test showed all the gravity conditions had a significant effect on upper lumbar curvature (χ25) = 31.032; *p* < 0.001). However, no effect at the lower sensor was observed on lumbar curvature (χ25) = 8.74; *p* = 0.120). Post-hoc analysis revealed a significant increase in distance in the upper sensor during µg (*p* = 0.001), lunar (*p* < 0.001), and Martian (*p* = 0.017) hypogravity conditions.

All mean values and post-hoc analysis results are shown in Table 1 and Table 2.

**TABLE 1:** Mean (±SD) spinal stiffness, normalized muscle activity, and lumbar curvature during Earth, Lunar, Martians, micro (µg), and hypergravity conditions.

	Micro-g	Lunar-g	Mars-g	Earth-g	Hyper-g-1	Hyper-g-2
	Mean ± SD	Mean ± SD	Mean ± SD	Mean ± SD	Mean ± SD	Mean ± SD
Spinal Stiffness (N)						
	73.94 ± 5.81	74.94 ± 5.43	76 ± 6.56	69.26 ± 10.07	67.48 ± 10.66	64 ± 9.69
**Muscle activity**	(#)	(#)	(#)	(#)	(#)	(#)
Erector spinae	0.91 ± 0.63	0.93 ± 0.51	0.99 ± 0.68	0.96 ± 0.69	1.00 ± 0.64	1.18 ± 0.54
Multifidi	1.18 ± 0.90	1.17 ± 0.78	1.15 ± 0.96	0.94 ± 0.76	1.17 ± 0.99	1.41 ± 1.07
Transversus abdominis	0.54 ± 0.27	0.51 ± 0.24	0.53 ± 0.29	0.97 ± 0.59	1.73 ± 1.05	1.58 ± 0.96
Iliopsoas	0.98 ± 0.46	0.96 ± 0.40	0.83 ± 0.36	0.91 ± 0.36	2.08 ± 0.92	1.41 ± 0.60
Lumbar curvature (mm)						
Upper distance	73.53 ± 10.87	73.88 ± 9.80	69.29 ± 10.59	65.59 ± 11.45	65.20 ± 11.60	65.33 ± 11.22
Lower distance	76.06 ± 16.53	82.50 ± 13.16	80.03 ± 16.09	78.55 ± 15.52	79.86 ± 16.54	78.79 ± 16.33

^a^ = gravity; SD, standard deviation; N = newton; mm = millimeter; #, Root mean square standardized to the average of the preceding and subsequent Earth gravity states.

**TABLE 2:** Changes in spinal stiffness, muscle activity, and lumbar curvature under the different gravitational conditions.

	Earth-g − micro-g	Earth-g − lunar-g	Earth-g − Mars-g	Micro-g − lunar-g	Micro-g − Mars-g	Earth-g − hyper-g-1	Earth-g—Hyper-g-2	Hyper-g-1—Hyper-g-2
**Spinal stiffness N)**								
*Z*	−2.118	−2.118	−2.667	−0.628	−1.255	−2.510	−0.981	−2.510
*p*	<0.034*	<0.034*	0.008*	0.530	0.209	<0.012*	<0.003*	<0.012*
∆%	+8%	+9%	+10%	+0.4%	+1.8%	- 5%	- 7%	−3%
**Muscle activity**								
Erector spinae								
*Z*	−0.459	−0.375	−0.356	−0.255	−0.051	−1.067	−1.956	−1.689
*p*	0.646	0.721	0.722	0.799	0.959	0.286	0.050	0.091
Multifidi								
*Z*	−0.978	−01.689	−1.956	−0.445	−0.356	−2.934	−2.943	−1.334
*p*	0.238	0.091	0.050	0.657	0.722	0.021	0.003^a^	0.182
Transversus abdominis								
*Z*	−2.756	−2.223	−2.667	−0.533	−0.445	−2.934	−2.934	−1.156
*p*	0.006^a^	0.026	0.008^a^	0.594	0.657	0.003^a^	0.003^a^	0.248
Iliopsoas								
*Z*	−1.156	<0.001	−1.511	−0.622	−1.423	−2.934	−2.845	−2.934
*p*	0.248	1.000	0.131	0.534	0.155	0.003^a^	0.004^a^	0.003^a^
**Lumbar curvature (mm)**								
Upper distance								
*z*	−2.666	−2.666	−2.429	−0.178	−0.059	−0.770	−0.652	−0.178
*p*	0.008^b^	<0.008^b^	0.015^b^	0.859	0.953	0.441	0.515	0.859
Lower distance								
*z*	−2.134	−0.533	−0.089	−1.007	−2.490	−1.334	−0.089	−1.511
*p*	0.33	0.594	0.929	0.314	0.013	0.182	0.929	0.131

N = newton; mm = millimeter; Wilcoxon signed ranks test,

^*^ *p* < 0.05; significant with Bonferroni correction.

^a^ *p* < 0.0125) and

^b^ *p* < 0.025, ∆% = change in percent.

## 4 Discussion

This study revealed a similar increase in spinal stiffness in a flattening of the upper lordosis, and thus spinal motor control modulation under lunar and Mars hypogravity conditions as observed in µg. Greater reduction in spinal stiffness were observed during the second hypergravity phase compared to the first. Hypergravity resulted in higher muscle activity and decreased gravity resulted in a flattening of the upper lumbar curvature.

### 4.1 Hypergravity

Confirming previous results from parabolic flight studies showing a change in spinal motor control strategy in hypergravity, this study (Swanenburg et al., 2018; Swanenburg et al., 2020) and a ground-based study showing a decrease in spinal stiffness in 100 participants carrying an axial load on their shoulders equal to 50% of their body weight showing (Hausler et al., 2020). A possible explanation for this decrease in spinal stiffness during hypergravity might be that the additional axial loading leads to activation of the abdominal muscles, which in turn may lead to a load shift away from the spine and direct transfer of the load to the thoracic cage and pelvis (Bergmark, 1989). This results in a de-loading of the spine and a decrease in spinal stiffness (Swanenburg et al., 2020). Glaus et al. (2021) concluded that the decrease in stiffness observed under large additional loads reflects a change in spinal motor control (Glaus et al., 2021).

The lower spinal stiffness observed during the second hypergravity phase suggests that there are residual effects of the μg phase. An increase in iliopsoas and transversus abdominis muscle activity in the second hypergravity phase compared with the first hypergravity phase was also observed. One possible explanation is that the difference between Earth’s gravity and hypergravity is smaller than the change from μg to the second hypergravity, with an extreme change leading to more extreme reactions. These results should be viewed with caution, as there were fewer useable measurements during the second hypergravity phase than the first due to the non-perfect pull-up slope of the airbus.

### 4.2 Decreased gravity

A similar increase in spinal stiffness, and thus spinal motor control modulation was observed during lunar and Martian hypogravity in addition to μg. This was not hypothesized but suggests that there may be a gravitational threshold including lunar and Martian hypogravity below which similar modulation is induced. In fact, this finding is consistent with the observation that axial load greater than 45% of body weight was required to modulate spinal motor control strategy (Glaus et al., 2021).

During µg, a decrease in transversus abdominis muscle activity, with no change in psoas muscle activity was observed, similar to that observed in previous studies (Swanenburg et al., 2020). The psoas muscle results are consistent with observations in long-duration space missions (LeBlanc et al., 2000; Chang et al., 2016). The psoas muscle, unlike all other muscles, does not degenerate during long-duration space missions it seems to be constant active (Chang et al., 2016). However, constant activation with no phases of relaxion of the psoas muscle might lead to muscle fatigue and lumbar spinal pain (Johnston et al., 1998; Barker et al., 2004; Swanenburg et al., 2020). On Earth psoas activation is often relieved by sitting (Bachrach, 1988), which is not possible during spaceflight. However, relaxation of the psoas muscle can also be achieved through adoption of the fetal position, which some astronauts have reported to amilorate LBP (Thornton et al., 1977; Penning, 2000). In fact, the psoas may play an important role in the acute development of LBP in astronauts in the beginning of their mission (Swanenburg et al., 2018; Swanenburg et al., 2020). This study, the muscle activity of multifidi increased during microgravity compared to earth gravity, but not significantly. Unlike the 2020 Study (Swanenburg et al., 2020), there were fewer measurement points since we also measured muscle activity on Lunar and Mars. The reduced number of measurements could explain the absence of a significant decrease in muscle activity.

#### 4.2.1 Spinal curvature

In this study, the upper lumbar distance sensor recorded a significantly larger distance between the backplate and lumber spine during µg, lunar, and Mars hypogravity, whereas the distance measures of the lower sensor did not change. These results indicate a flattening of the spine with the greatest change in the upper lumbar spine. A flat spine curvature has also been observed in astronauts returning from space missions (Burkhart et al., 2020). A flattened lower lordosis was also observed after a bed rest study, which also showed that the upper lumbar spine became more lordotic (Belavy et al., 2011). A more detailed picture would be useful to explain these adaptations Measurements at additional measurement points along the spine are needed for a more accurate assessment of spinal curvature.

During a long space missions, a decrease in muscle activity and a degeneration of muscle mass can be observed in most of the back muscles that work mainly against gravity (LeBlanc et al., 2000; Chang et al., 2016). But unlike other studied muscles of the lumbar spine, this does not apply to the psoas muscle; neither does its activity decrease nor does it degenerate with prolonged exposure to a microgravity environment (Andreoni et al., 2000). It is possible that the psoas muscle, which can adjust its activity to the current degree of lordosis in an upright position, stabilizes the lower lumbar spine (Penning, 2000). By adjusting its activity to the current degree of lumbar curvature it could have stabilizing effect of the lower lumbar spine in a microgravity environment (Penning, 2000).

If this assumption of a constant activation of the psoas muscle is correct, which in turn might lead to psoas muscle fatigue and pain in the lumbar region (Johnston et al., 1998; Barker et al., 2004; Granata et al., 2004). This psoas complaints could be alleviated on Earth by sitting sand therefore relaxing the psoas muscle (Bachrach, 1988). However, sitting is not possible in a microgravity environment. Nevertheless, a similar relaxation of the psoas muscle can be induced in microgravity by adopting the fetal tuck position. Astronauts reported by adopting the fetal tuck position can bring about relief from pain (Thornton et al., 1977; Penning, 2000). Based on these considerations and observations, we have proposed that the psoas muscle has a central role in the development of back pain in astronauts (Swanenburg et al., 2018).

The constant activation of the psoas muscle and the resulting adaption of the upper lumbar spine during reduced gravity conditions might also be an explanation for the fact that injuries to the IVD tissue in astronauts are found in the upper part of the lumbar spine. In the start of a space mission, the tissue of the IVD and ligament of the astronauts has the quality for a safe movement on earth. Without a load, the earth quality IVD tissue can adapt in a μg condition without risk of injury. After 6 months in space, when the astronauts return to Earth, the IVD tissue must adapt back to the earth’s gravity. But this time the IVD tissue and ligaments have degenerated and has only the quality suitable to the μg. This degeneration of IVD tissue has been confirmed by MRI examinations following long duration spaceflight (Chang et al., 2016; Bailey et al., 2022) This degeneration of IVD tissue can lead to an increased risk of IVD tissue damage of the upper lumbar spine and may explain why 60% of all astronaut spinal injuries occur in the upper lumbar spine (Bailey et al., 2022).

Another explanation for the acute change in spinal motor control could be the flattening of the spine found in this study. However, the exact location and extent of spinal flattening are unknown. Nevertheless, the acute change in lumbar spine curvature was minimal, and the passive structures of the spine remained within the neutral zone throughout the measurement period. Consequently, the influence of the passive structures on the stiffness of the spine is considered insignificant under both reduced gravity and increased gravity conditions. Nevertheless, this question has potential for future evaluation.

The acute changes we found here in this study are to be compared to the chronic adaptations observed in the astronauts. The spine of an astronaut flattens during long space missions in a µg environment (Chang et al., 2016) and the muscle activity in most muscles (except the psoas) are less active (Andreoni et al., 2000). We do not know about spinal stiffness because it is not measured during a space mission. However, preliminary data show that astronauts exhibit reduced spinal stiffness after a 6-month space mission. They respond in the same way as if they were carrying extra axial weight.

### 4.3 Possible consequences for orbital and lunar operations

The sensitivity of spinal stiffness to gravitational changes and a potential link to vertebral kinematics (Breen et al., 2023) suggests that its evaluation is warranted during spaceflight, but also in response to ARED use, and candidate spinal deconditioning countermeasures such as axial-loading provided by SkinSuit, as such loading has been suggested to promote spinal control on Earth (Rathinam et al., 2013). Recently, the Mk VI SkinSuit (Stabler et al., 2017), devised to provide ≈0.2gz axial loading ameliorated spinal elongation and back pain by ≈ 50% induced by 8-h HBF by ≈ 50% (Green et al., 2023), as well as restoring lumbar mobility and lordosis following 4 h HBF (Breen et al., 2023).

The data above also suggests that the spine is highly sensitive to gravity transitions, but that Lunar and Martian gravity are below that required for normal modulation of spinal motor stabilization strategy. This is critical as crew will in the near future be required to perform repeated Lunar surface operations including frequent placing and carriage of payloads and the use of tools for geological/biological sampling (Crawford et al., 2012). If crews have sub-optimal spinal motor stabilization strategies the risk of a critical fall, LBP and/or IVD pathology all of which could be mission critical could be unacceptably elevated. Thus, complete definition of the underlying pathophysiological mechanisms is required to help define optimized spinal countermeasures (Green and Scott, 2018) compatible with exploration missions (Scott et al., 2019). Where it is important to distinguish between short time effects (beginning of a mission) and longtime effects (after a mission).

### 4.4 Limitations

There were artifacts in data recorded using ultrasonic distance sensors that were difficult to clean and thus the derived results could be biased. Nevertheless, for some participants a repetitive pattern in accordance with the course of parabolas was apparent from the data. These results should thus be treated as hypothesis generating rather than a confirmatory result. Furthermore, this study only employed two distance sensors to determine the upper and lower lumbar curvature. A greater density of sensors is required to assess the effect of gravity changes more comprehensively upon spinal curvature.

Measurements in the cranio-caudal direction could be an important proxy measurement of spinal motor control with respect to the amortizing spring properties of the spine. These measurements may have potential for future studies.

In this study, we did not conduct a systematic survey regarding the volunteers’ subjective feelings during the parabolic flight. However, all subjects were asked what they experienced during the parabolic flight and how they felt. None of the subjects reported pain at any point and to our knowledge this has not been reported in any previous Parabolic Flight campaign, suggesting that the effects underlying spatial adaptation take longer to manifest. Such as one of a dry immersion to simulate microgravity study (Treffel et al., 2017).

## 5 Conclusion

The study revealed that microgravity, lunar gravity, and Martian gravity conditions led to comparable increases in spinal stiffness. Additionally, there was a decrease in transversus abdominis muscle activity, while psoas muscle activity remained unchanged. Lower gravity resulted also induced acute flattening of the upper, but not lower lordosis. Hypergravity resulted in a decrease in posterior-to-anterior spinal stiffness and higher muscle activity for m. Psoas and m. Transversus abdominis. These observations indicated a modification in the strategy for stabilizing the spine’s motor function under different gravitational conditions. Given the effect of microgravity, and both lunar and Martian hypogravity are similar—it appears that normal vertebral stiffness control may operate within a specific g window. The absence of a typical spinal motor stabilization strategy in Lunar and Martian gravity conditions has the potential to increase the risk of lower back pain (LBP) and/or intervertebral disc (IVD) issues unless suitable countermeasures are put in place.

## Data Availability

The raw data supporting the conclusion of this article will be made available by the authors, without undue reservation.
